# Homœopathic Instructions for Families with Reference to the Cholera

**Published:** 1866-02

**Authors:** 


HOMŒOPATHIC INSTRUCTIONS FOR FAMILIES WITH REFERENCE TO THE CHOLERA.

At a meeting of the Hahnemann Academy of Medicine, held July 16, 1854, the Committee on Cholera reported the following instructions for the domestic management of this disease:

1. Avoid crowded assemblies and crowded sleeping apartments, and as much as possible shun the presence of filthy persons, for the disease is mostly developed in crowded dwellings, ships, prisons, camps, &c.

2. Observe cleanliness of person and enjoin the same upon your household.

3. Dwellings—especially the sleeping apartments—should in all cases be thoroughly ventilated.

4. Pursue your ordinary course of diet, observing some moderation as to vegetables and fruits. Night meals are to be avoided. Regularity in the hours of eating is very desirable. Alcoholic drinks are objectionable, the intemperate being particularly liable to this disease. Ice-water and ices should be used with extreme moderation. Articles of diet known to disagree with the regular action of the bowels should be most scrupulously avoided.

5. Avoid mental or bodily excitement or fatigue. Keep the person warmly clad.

6. Cathartics and laxatives must be wholly avoided. No means should be taken to remove constipation, except such as are prescribed by a physician. The use of laudanum, opium, or cholera mixtures of any kind is hazardous.

7. It is better to take no medicine as preventative of cholera, but the slightest derangement of the bowels should be met by appropriate treatment.

8. Should there be oppression or sickness at the stomach, shiverings or dizziness, with or without relaxed bowels, *Ipecac* of the second or third trituration or dilution, may be taken every two or three hours.

9. If there be watery looseness of the bowels, with or without nausea, pain or cramps, take one drop of *Veratrum*, first dilution, every half hour or hour.

10. If the diarrhœa should become profuse, with or without pain or vomiting, discharges very frequent, being watery or resembling rice-water, with or without cramps, coldness, and blueness, with rapid sinking, take one or two drops of the spirits of camphor every five or ten minutes until reaction takes place.

From the moment the diarrhœa becomes urgent, the patient should go to bed and be well wrapped with blankets. Bottles of hot water should be applied to the feet, and medical aid at once be summoned. No external use of camphor is advisable while other remedies are employed.

Published by order of the Hahnemann Academy of Medicine, New York.

It may seem out of time and place, to fill two whole numbers of this Journal with remarks on the Cholera, in the winter time, when it has never prevailed in our country during that season, and may not appear among us in the next ten years. But, in all probability it will visit us next summer; such is the apprehension of scientific men; and such has been its usual course; hence, it is thought advisable to consider the subject in time, especially as there are four facts of inconceivable importance, which ought to be known to every human being liable to an attack.

First. The primary cause of Cholera is in the atmosphere, but that primary cause may be said to be almost as incapable of causing an individual attack of Cholera, as powder is incapable of detonation, without the application of a spark; or as tubercle is incapable of causing common consumption of the lungs without the application of causes which soften it, this softening being the actual disease.

Second. The immediate cause of epidemic cholera, that is, the thing which excites cholera in a choleric atmosphere, is an emanation from the surface of the earth, in localities where vegetable matter and house offal is in a state of decomposition, with certain exceptions.

Third. There is scarcely any ordinary disease which is so easily and so certainly prevented, as cholera.

Fourth. It is difficult to remember the name of any disease, which is so easily and so infallibly and so perfectly cured, as epidemic cholera, if prompt measures are taken at the very first indications of its approach; this Fourth head has been fully presented in the preceding pages; it is the third, to which special, present and personal attention is directed, that is, the easy preventability of cholera, if timely and possible efforts be made to that end.

During the last prevalence of cholera,it made its appearance in only one house in a large district of houses, which had been closely inspected and cleansed. On closer investigation, a large heap of house and kitchen offal, the apparent accumulation of years, was found in a dark corner of the cellar.

In one of the larger cities in the northern part of the State of New-York, the cholera made its first,its most malignant and long continued onset, on the line of a street which was in process of being dug up for some necessary improvement, a filling having been made there, and some years before.

While the Erie Canal was in process of construction, and also again in building the Hudson River Railroad, destructive fevers attacked the workmen engaged in those parts of the line where the fillings up of low places with leaves, rotten wood, &c., had gone on for great lengths of time. The practical conclusions which force themselves on the most common minds is that, 1st, Every householder owes it to himself, to his family, to his neighbors, and to the community in which he resides, to have his house, from cellar to garret, from the street curb to the rear line of his lot, most scrupulously cleansed, by sweeping, washing and whitewashing. 2d, Every man who has any authority in city or town government, should consider himself bound by the oath of office, and by every consideration of humanity, to give himself no rest, until every street, alley, close gutter and sewer, is placed in a state of as perfect cleanliness as possible, and kept so, until the frosts of next season come. 3d, These cleansings should be done now, in February and March, because, if put off until warm weather, the very effort necessary to the removal of filth, will only tend, in the essential nature of things, to hasten the appearance of the disease, to increase its malignity, and to extend the time of its devastations ; because, the suns of spring and summer the sooner warm into life and intensify the viperic and malignant influence, which, in its remorseless tread, wrecks so much of human happiness and desolates so many hearth stones.

"A Father's Letters to his Daughter," by Robert West.

"Effie Morrison or the Family of the Redbraes," a narrative of truth, by the author of Allan Cameron, &c., 157 pp.

A dollar and a quarter will purchase the five, and would be a valuable, instructive present for any child or friend.

THE AMERICAN TRACT SOCIETY, 28 Cornhill, Boston, and 13 Bible House, New-York, present to the christian public for reading in their own families and for distribution to friends, to kindred, to employees and to neighbors, the following,

"Michael the Miner," a Hungarian story, 120 pp.

"The Lolster Boy; or the Son who was a heaviness to his Mother," by the Author of the Fisher Boy, 120 pp.

"Kate Woodman, or the heart revealed," by Alice A. Dodge, author of The Way to the Cross, &c., pp. 229.

"The Daughters of the Prairie," 301 pp, a narrative of great interest and highly instructive to young and old:

"Frank Victory and the Nevers," 144 pp., and "The Fisher Boy, or the Son who made a glad Father," by the author of the Casket Library, 107. "The Cup Bearer," tinted paper with illustrations, will feed and delight any Christian heart.

"Victor's Stories for Boys and Girls," by the writer of Uncle Paul's Stories, 144 pp. Five dollars will buy all these publications; there is enough reading in them to profitably engage the attention for weeks and even months, for they will bear repeated readings, and then they might be distributed here and there among neighbors, not only to benefit and elevate them, but to excite a taste perhaps for such reading, thus increasing the patronage of the Society which has done so much in years past, and will do more in years to come, to furnish instructive and elevating books for our whole country.

"EVERY SATURDAY," Vol.1, No.1, published weekly by Ticknor & Fields, 124 Tremont St. Boston, Mass. A journal of choice reading, selected from foreign current literature. Single Nos. 10 cts.; five dollars a year in advance. This first number has articles on Precious Stones, The Spectral Rout, Tupperides, Who was Frederic Robertson, More of Our Dogs, Our Brown Passenger, An Apology for the Nerves, Land Martins. Whoever reads the first number will want to be a subscriber.

"Every Saturday" contains 28 pages 8vo. of reading matter, and will be put at four dollars a year to those who take any other of Ticknor & Field's periodicals.

"The Mother's Assistant and Child's Friend," published monthly, by C. H. Pearson & Co. at 21 Cornhill, Boston, $2.50 a year, is admirably adapted to the wants of every family, and deserves a wide patronage; the popular and very able writer, Rev. Wm. M. Thayer, will be a frequent contributor to its pages.

"The Richmond Medical Journal," is published at Richmond, Virginia, at $5, and is issued quarterly and is ably edited.

THE MOTHER'S ASSISTANT. This is the growing Magazine, the best as it is the oldest of the kind. Wide-awake and up to the times, sensible, pithy, suggestive, genial, lively, practical, and of sterling religious influence, few families can afford to do without it. We are happy to learn that it is meeting with the success it so richly merits. It is the accredited organ of the "Union Maternal Association of Boston," and is greatly to their credit. What cannot a band of noble ladies accomplish under suitable leadership? Evidently there is a "born editor" at the helm, or this Monthly could not be what it is. Published by H. Pearson & Co., 21 Cornhill, Boston. $1.50 pr. annum; $1 to clubs.

VITAL GODLINESS: A Treatise on Experimental and Practical Piety: by Rev. Wm. S. Plummer D.D., LL.D,; one vol. 12mo, 610 pp. $1; just published by the American Tract Society, 150 Nassau Street, New-York; sent by mail to any part of the United States for $1.25. This excellent work embodies the fruits of the venerable author's long and diligent study of religious truth, and of his remarkable personal and ministerial experience. It is a valuable help in Chris-tian life.

HOUSE VENTILATOR and Air Purifier, patented by A. S. Lyman, New-York, is deservedly attracting public attention.

PHOTOGRAPHIC.—G. W. Hope, 233 Broadway, New-York, makes as fine pictures as the best city artists, at greatly reduced rates. He merits public patronage.

HEALTH TRACTS, 236 in number, with a splendid steel engraving of the Editor of Hall's Journal of Health, is sent post paid by P. C. GODFREY, 831 Broadway, for $2.50.

**WANTED TO GIVE** to any person who will take the time and trouble to procure Sixty paying subscribers to Hall's Journal of Health for 1866 at $1.50 a year, the choice at the establishment of Wheeler & Wilson, 625 Broadway, New York, of one of their best Sewing Machines. which are sold for cash, at Fifty-Six Dollars each, and the same in proportion for any other priced Machine up to $195. The machine offered will sew all kinds of fabrics and is the cheapest machine manufactured. Specimen number sent post paid for ten cents. P. C. Godfrey, 831 Broadway, New York.

